# Atomistic simulations of dislocation mobility in refractory high-entropy alloys and the effect of chemical short-range order

**DOI:** 10.1038/s41467-021-25134-0

**Published:** 2021-08-11

**Authors:** Sheng Yin, Yunxing Zuo, Anas Abu-Odeh, Hui Zheng, Xiang-Guo Li, Jun Ding, Shyue Ping Ong, Mark Asta, Robert O. Ritchie

**Affiliations:** 1 Materials Sciences Division, Lawrence Berkeley National Laboratory, Berkeley, CA USA; 2 Department of Materials Science and Engineering, University of California, Berkeley, CA USA; 3 Department of NanoEngineering, University of California San Diego, La Jolla, CA USA; 4 Center for Alloy Innovation and Design, State Key Laboratory for Mechanical Behavior of Materials, Xi’an Jiaotong University, Xi’an, China

**Keywords:** Mechanical engineering, Structural materials, Atomistic models

## Abstract

Refractory high-entropy alloys (RHEAs) are designed for high elevated-temperature strength, with both edge and screw dislocations playing an important role for plastic deformation. However, they can also display a significant energetic driving force for chemical short-range ordering (SRO). Here, we investigate mechanisms underlying the mobilities of screw and edge dislocations in the body-centered cubic MoNbTaW RHEA over a wide temperature range using extensive molecular dynamics simulations based on a highly-accurate machine-learning interatomic potential. Further, we specifically evaluate how these mechanisms are affected by the presence of SRO. The mobility of edge dislocations is found to be enhanced by the presence of SRO, whereas the rate of double-kink nucleation in the motion of screw dislocations is reduced, although this influence of SRO appears to be attenuated at increasing temperature. Independent of the presence of SRO, a cross-slip locking mechanism is observed for the motion of screws, which provides for extra strengthening for refractory high-entropy alloy system.

## Abstract (web summary)

Refractory high entropy alloys hold big promise for elevated-temperature applications. Here the authors investigate the influence of short-range order on the mobility of dislocations in high-entropy alloys by large-scale molecular dynamics simulation based on a machine-learning interatomic potential.

## Introduction

Metallic alloys containing multiple principal alloying elements, which are known as high-entropy alloys (HEAs)^1–3^, have attracted extensive research interest in recent years with many studies focused on their microstructure, mechanical properties, and underlying deformation mechanisms^4–6^. For example, as a new class of structural materials, certain face-centered cubic (*fcc*) alloys, in particular CrCoNi-based HEAs, have been shown to possess improved strength and exceptional damage tolerance, especially at cryogenic temperatures^7,8^.

A second prominent class of HEA systems are refractory high-entropy alloys (RHEAs), which comprise mostly refractory elements and invariably crystallize as body-centered cubic (*bcc*) solid solutions; these alloys have been considered as promising candidate materials for elevated-temperature applications due to their exceptional resistance to softening and extremely high melting points^9–11^. Although many practical challenges remain in the processing of RHEAs due to their brittleness and oxidation susceptibility^12^, numerous RHEAs have been designed, fabricated, and assessed experimentally, with additional insight coming from computational approaches^13–17^. Specifically, to investigate the deformation of RHEAs, transmission electron microscopy (TEM) studies on RHEAs have shown a dominant role of screw dislocations with increasing plastic strain^18,19^, and slip activity on high-order-planes has been observed through in situ scanning electron microscopy experiments^16^; indeed, strong intrinsic lattice resistance has been generally found in these concentrated solid-solution alloys^19,20^. Theoretical models for strengthening in RHEAs by screw dislocations have been developed^21,22^, and atomistic simulations of dislocation properties using molecular dynamics (MD) and density-functional theory (DFT) calculations have been undertaken^23–27^. Recently, mesoscale models, such as a kinetic Monte Carlo model, have also been developed to investigate the strengthening in RHEAs^28^. However, compared to pure *bcc* metals, there are still limited studies on the unique deformation behavior of *bcc* RHEAs.

Although HEAs possess crystalline order, but compositional disorder, they are not necessarily random solid solutions for every atomic species^29–37^. Specifically, a feature of HEAs is the possibility of the presence of local chemical short-range order (SRO), which can affect defect motion and hence may influence mechanical properties^27,30,38,39^. In previous studies, SRO effects in *fcc* or *hcp* alloys have been found to be able to change the dislocation morphology, i.e., from wavy to planar, and may impact twinning^40,41^. Recent experimental results have provided direct evidence for the presence of SRO in certain *fcc* HEAs and its correlation with mechanical properties^39,42^. Moreover, for RHEAs, such as the MoNbTaW, numerical studies have revealed a significant energetic driving force for SRO^27,31,32,43^. The effect of such SRO is thus a relevant issue that requires further investigation for single-phase *bcc*-RHEA solid solutions.

To our knowledge, to date, there are no detailed investigations of the effects of SRO on larger-scale defect properties, especially dislocation mobility, for *bcc* RHEAs. This situation provides the motivation for this study. Accordingly, our objective here is to employ MD simulations, based on a machine-learning interatomic potential developed for this purpose, to investigate in detail the mechanisms of the movement of dislocations as a function of temperature in *bcc* RHEAs, focusing on the single-phase MoNbTaW system, with specific emphasis on the effects of SRO.

## Results and discussion

### Machine-learning interatomic potential

**Fig. 1:**
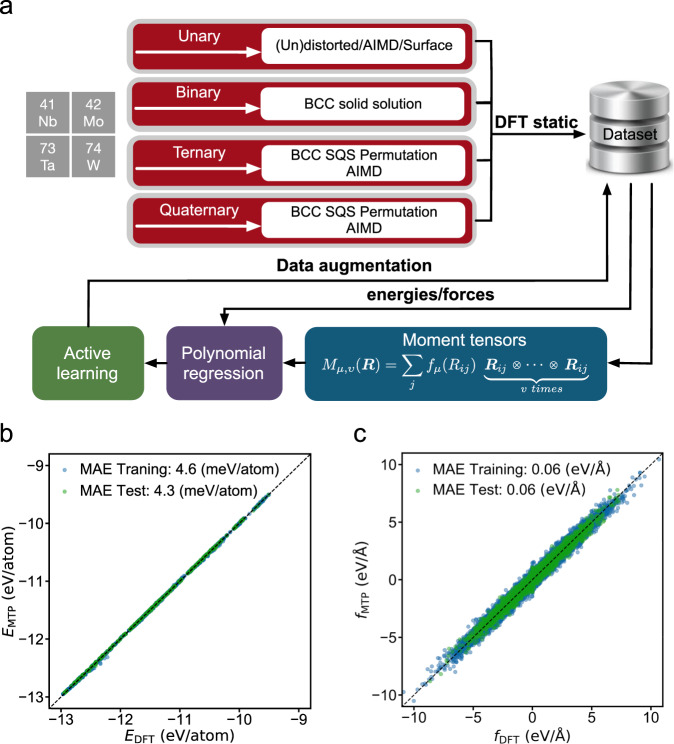
Machine-learning interatomic-potential development and evaluation. **a** Flowchart of constructing the moment tensor potential (MTP) for the NbMoTaW RHEA. Parity plots of the MTP prediction on **b**, energies and **c**, forces for training and test data are shown.

In this section, we discuss the results of the development of the interatomic-potential model used to enable the application of MD simulations of dislocation properties in *bcc* MoNbTaW. We employ the general framework of machine-learning (ML)-based approaches, which have emerged recently as a systematic framework to develop potentials with accuracies near that of the underlying DFT calculations for elemental and multicomponent systems^24,44–46^. Here, using training data reported previously^24^ for elemental, binary, ternary, and quaternary systems in Nb–Ta–Mo–W, we developed a potential for the MoNbTaW RHEA based on the moment tensor potential (MTP) formalism, which has been found to feature an excellent balance between accuracy and computational cost^46^. The flowchart for constructing the MTP is shown in Fig. 1a with the methodological details provided in “Methods”. The basic materials property predictions of the MTP model are provided in Supplementary Table 1. Figure 1b, c shows a comparison of the DFT- and MTP-predicted energies and forces for both training and test sets. While the mean absolute errors (MAEs) of energies of 4.6 and 4.3 meV atom^−1^ for respective training and test data are similar to those reported for previously developed potentials^24^, the MAEs of forces were decreased by half using the present implementation of MTP, compared to the previous spectral neighbor analysis potential (SNAP)^24^. We further performed tenfold cross-validation and the average MAEs for energies and forces of the validation data were 4.4 meV/atom and 0.056 eV/Å, respectively, in line with Fig. 1a and b. In addition, since the current work aims to investigate dislocation properties through MD simulations, we have also undertaken further validation studies of the MTP for dislocation energetics. Specifically, we consider the distribution of excess energies for supercells with a dislocation dipole, as the dislocation cores are moved to different local environments within the cell, as calculated in recent DFT calculations for systems with varying degrees of SRO^27^. The distribution of dislocation dipole energies obtained with the MTP is found to be in excellent agreement with these previous DFT results, as shown in Supplementary Fig. 1. These results suggest that the MTP provides an accurate model for the variation in dislocation core energies as a function of the local environment, motivating its use in the current MD studies of dislocation mobilities.

### Local chemical short-range order in MoNbTaW RHEA

To enable investigation of the effect of SRO on dislocation mobility, we develop two simulation cells that can be used for such simulations in MD, one corresponding to a random solid solution (RSS), and another corresponding to an equilibrium state of SRO at a temperature of 800 K (this temperature is chosen as one where SRO is relatively strong but above that associated with long-range ordering transitions). To equilibrate the SRO state, we apply a hybrid MD/Monte Carlo (MD/MC) approach. Details of the simulations and the nature of the equilibrium SRO are described in “Methods” and Supplementary Note 1.

We note that in the studies of dislocation mobility at different temperatures presented below, we compare results for both RSS and systems with SRO equilibrated at 800 K. The purpose of this comparison is to explore the effect of SRO on the mechanisms underlying dislocation motion in a single-phase homogeneous solid-solution alloy without long-range order; the SRO state at 800 K is chosen as a representative configuration where SRO is pronounced. The current simulations are intended to address the situation where dislocation glide is very fast relative to the diffusion hopping timescale, such that changes in ordering and composition around the dislocation are not considered. Thus, in this scenario, we are simulating the effect of SRO derived from annealing the sample at one single temperature (800 K), on the subsequent motion of dislocations over a range of deformation temperatures, at fast timescales over which the state of SRO does not change and is not altered around the moving dislocation core.

### Screw-dislocation velocities and the influence of SRO

A screw dislocation line is created in both the RSS and SRO-simulation supercells. Shear stresses with a range of different values are applied at different temperatures to drive the motion of the screw dislocations and characterize their mobility (see “Methods”). It is worth mentioning that due to practical considerations of computation cost, the current work considers a periodic length along the dislocation line direction of ~15 nm. The simulations thus investigate the motion of dislocations with line lengths that are quite short compared with those characteristics of realistic experimental conditions and considered in theoretical models^22^. The velocities calculated in the current setup are expected to be dependent on length in the range of size employed in this work^47^. Thus, the focus in the present work is on understanding the intrinsic mechanisms underlying the motion of short dislocation segments, and the associated effects of SRO; detailed comparisons of the magnitude of such mobility would require the use of this information in theoretical models that can be extrapolated to larger line length scales.

**Fig. 2:**
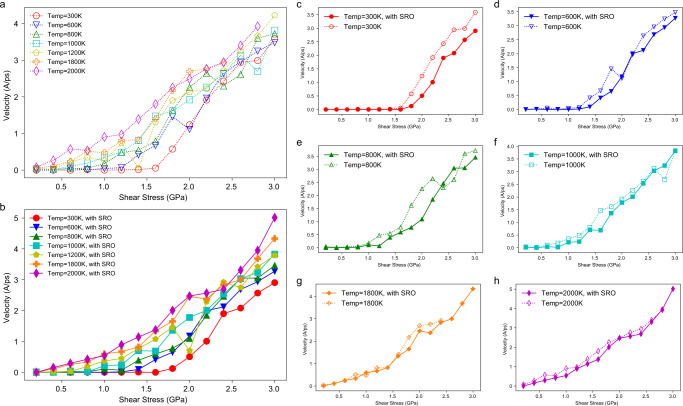
The effect of SRO on the velocities of screw dislocations. **a** Velocities of screw dislocations in the RSS sample as a function of applied shear strain. **b** Velocities of screw dislocations versus shear stress in the sample with local chemical SRO equilibrated at 800 K. **c**–**h** Detailed comparisons of the influence of SRO at different temperatures from 300 to 2000 K. Lines between scattered data are guides to the eyes and do not correspond to fits of theoretical models.

Figure 2 shows the velocities of screw dislocations as a function of the applied shear stress in cells with and without SRO at various temperatures. In Fig. 2a, the dashed lines represent the velocity vs. stress data for a screw dislocation in the RSS sample under applied stresses ranging from 0 to 3.0 GPa, at temperatures from 300 to 2000 K, whereas the solid lines in Fig. 2b represent the corresponding results for a screw dislocation in the sample with SRO under the same conditions. The results show that, at low temperatures, the screw dislocation does not move under low values of the applied stress within the simulation timescale. When the stress is increased above some critical level, we can observe the initiation of screw dislocation movement at a velocity that increases with the magnitude of the applied shear stress. As the temperature increases, the critical stress to initiate screw dislocation glide is progressively reduced. When the temperature reaches 1800 or 2000 K, even at the lowest applied stress of 200 MPa in our simulation, screw dislocation glide can be clearly observed within the simulation timescale. Overall, the results demonstrate that screw dislocation glide in the MoNbTaW RHEA exhibits strongly temperature-dependent behavior.

Figure 2c–h compares the screw-dislocation velocities with and without SRO at different temperatures. At 300 K (Fig. 2c), the influence of SRO is clearly apparent; based on the shift in the velocity curve, we can estimate the extra strengthening from SRO to be ~180 MPa. We can attribute this SRO effect to be associated with the break-up of local chemical order which creates diffuse antiphase boundaries (DAPBs)^48^. When the screw-dislocation glides in a system containing SRO, the order will be partially destroyed and a DAPB will be created along the glide plane, with an energy cost per area *γ*_*DAPB*_. A classical estimation of this strengthening effect of SRO by Fisher^48^ can be written as: $\triangle \tau ={\gamma }_{{DAPB}}/b$, where $\triangle \tau$ is the stress increment due to SRO and $b$ is the Burgers vector. Using the value of the ${\gamma }_{{DAPB}}$ from Supplementary Fig. 2, the stress increment based on Fisher’s relationship is ~130 MPa, which is comparable to the shift in the velocity curves from the MD simulations in Fig. 2c, d at temperatures of 300 and 600 K. However, in this model the strengthening effect of SRO is athermal and should also be observed at higher temperatures, contrary to what is observed in the current simulation data.

The influence of SRO at higher temperatures is illustrated in Fig. 2e, f, for temperatures ranging from *T* = 800 to 2000 K. There is a clear attenuation of the SRO effect in these figures as the temperature is increased. Indeed, when the temperature reaches 2000 K, the dashed and solid lines in Fig. 2f, respectively, representing the screw dislocation mobility in the RSS and SRO samples, essentially overlap. A theoretical framework for interpreting the effect of temperature on the level of SRO strengthening is presented below.

### Screw-dislocation glide mechanism and the strengthening through cross-slip locking

**Fig. 3:**
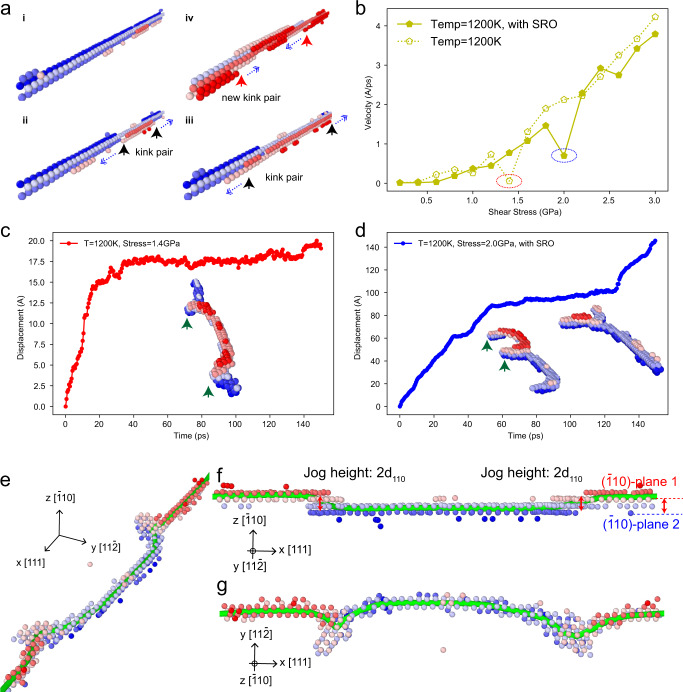
Kink nucleation mechanism and the observation of cross-slip locking. **a** Snapshots of atomic configurations along the screw dislocation, illustrating kink nucleation during glide. (i) The initially straight screw dislocation line. (ii) A kink pair nucleates (indicated by the black arrows) and migrates along the dislocation line (dashed arrow). (iii) Kink-pair migration leads to the net motion of the screw dislocation line. (iv) Another kink pair nucleates (indicated by the red arrows) and migrates. Only the dislocation core atoms are shown and the atoms are colored by coordinates in the glide direction $[1\bar{1}2]$. **b** Simulation velocity vs. stress results for the screw dislocation at 1200 K; the circles show anomalous points yielding a significantly lower average velocity, associated with the formation of cross-slip locking. Details of the dislocation configurations are shown in (**c**, **d**). **c** Displacement vs. time curve of a screw dislocation under a shear stress of 1.4 GPa at 1200 K in the random sample. The inset shows the formation of cross-slip locking during screw dislocation glide. **d** Corresponding displacement vs. time curve of a screw dislocation under a shear stress of 2.0 GPa at 1200 K in the SRO sample. The inset figure shows the formation and unlocking of cross-slip locking during screw dislocation glide. **e** Perspective view of the dislocation line with cross-slip locking. The blue region and red region are on slip planes with different *z* coordinates. **f** Dislocation line with cross-slip locking viewed from the *y* direction. The red segment of the dislocation line in on plane 1 and the blue segment is on plane 2; the two segments are connected by two jogs with a height of 2*d*_110_. **g** Dislocation line with cross-slip locking viewed from the *z* direction. In **e**–**g**, only the atoms around the screw dislocation line are shown and are colored by the coordinates in the *z* direction. The green line represents the <111> screw dislocation line.

An analysis of the atomic configurations during screw dislocation glide reveals that the dominant mechanism underlying dislocation motion for the dislocation lengths and timescales probed in the lower-to-middle temperature and stress regimes, corresponds to thermally activated kink-pair nucleation and migration^49^, with a strong temperature-dependence similar to that seen in pure *bcc* metals. Figure 3a(i–iv) displays a sequence of snapshots from the simulations highlighting kink-pair nucleation and migration along the dislocation line.

From an analysis of the velocity data for various temperatures and stresses from all simulations, it is found that certain simulations show anomalously low average dislocation velocities compared to other data. For example, in Fig. 3b the velocities at 1200 K in both RSS and SRO samples exhibit two data points with very low-velocity values, as highlighted by the dashed circles. Figure 3c shows the displacement vs. time results for a screw dislocation in the simulation with 1.4 GPa shear stress in the RSS sample, where it can be seen that the dislocation moves initially at a constant velocity via the kink-pair nucleation and migration mechanism; however, at a certain point, this glide is arrested due to the formation of a locking mechanism such that no further glide is observed.

In *bcc* metals, cross-kinks are formed when multiple kink pairs nucleate on the same dislocation line but on different slip planes; breaking free from such cross-kink pinning sites requires higher stress or thermal activation. This phenomenon thus provides extra strengthening and can lead to debris such as vacancies, interstitials, and prismatic loops, as described in theoretical models^22^, kinetic MC simulations^50^, and directly observed in TEM experiments^20^. However, the cross-slip jogs observed here are somewhat more complex than these previously defined cross-kinks, as shown by the insets in Fig. 3c. Specifically, this figure displays the screw dislocation line with periodic boundary conditions where the atoms are colored by the height of the slip planes in $[\bar{1}10]$. In the inset, one segment of the dislocation line has moved to the upper slip plane by cross-slip, represented by the red color, and two jogs are created at the intersections between the cross-slipped segment and the original blue segment, as shown by the two arrows in the inset figure. Details of the nature of this locking is shown in Fig. 3e–g. Extra stress is then required for the dislocation to break or move these jogs, as shown in Supplementary Fig. 3. In this case, the whole dislocation line becomes immobile (on the timescale of the MD simulation) due to the pinned jogs during the entire simulation time after they form.

In another simulation shown in Fig. 3d for the sample with SRO, where the shear stress is higher (2.0 GPa), we can observe the locking formation and self-unlocking during the simulation. Supplementary Movie 1 shows the details of this process. From the displacement vs. time curve in Fig. 3d, the dislocation first glides at a constant velocity and then becomes immobile due to the locking, as shown by the insets in the figure. Because of the high applied shear stress in this case, after some time the jogs become automatically unlocked such that the screw dislocation can resume gliding; as such, the breaking of such jogs provides extra strengthening for the RHEA.

From the theoretical model of Maresca and Curtin^22^, the frequency of such cross-kinks depends on the ratio of the kink energy and the solute-dislocation interaction energy; accordingly, the formation of cross-kinks and jogs (superkinks) would be expected to be easier in the RHEA concentrated solid solution as compared to pure *bcc* metals or dilute binary alloys. In addition, the intrinsic fluctuation in energy along the dislocation line and the rugged energy landscape for screw dislocation segments in RHEAs, which have been observed in previous DFT and MD simulations^25,27,51^, can further facilitate cross-slip along the dislocation line and cause the formation of jogs, which strongly implies that cross-kink and cross-slip locking plays a more important role in the deformation and strengthening of RHEAs, as compared to pure *bcc* metals.

### Phenomenological dislocation mobility model

The previous two sections have highlighted two strengthening mechanisms underlying the motion of screw dislocations— double-kink nucleation and glide, and cross-kink formation. Prediction of how these mechanisms will combine to determine material strength would require appealing to mesoscale models for longer dislocation lengths and longer timescales, which is beyond the scope of the current work. In this section, we consider further the effect of SRO on dislocation velocities arising exclusively from the double-kink mechanism, employing a phenomenological theoretical model for this mechanism, in comparison to the MD data which exclude the cross-kink mechanism. In other words, in this analysis we exclude the effects of the cross-slip locking and focus on the data associated with dislocation glide in the absence of such pinning events, to understand the temperature-dependent SRO effects associated with dislocation glide through the kink nucleation and growth mechanism.

Specifically, the MD results are analyzed within the theoretical framework developed by Po et al.^52^, which is extended to account for the effect of SRO on the free energy of kink-pair nucleation. In the low temperature and low-stress regimes, dislocation mobility is deemed to be governed by kink-pair nucleation and migration, with the Gibbs free energy for kink-pair formation, $\Delta {G}_{{kp}},$ written as:

1 $$\Delta {G}_{{kp}}=\Delta {H}_{{kp}}-T\,\Delta {S}_{{kp}},$$

where $\Delta {H}_{{kp}}$ is the formation enthalpy, and $\Delta {S}_{{kp}}$ is the corresponding entropy. The formation enthalpy is usually written as:

2 $$\Delta {H}_{{kp}}=\Delta {H}_{0}\left\{{\left[1-{\left(\frac{\tau }{{\tau }_{0}}\right)}^{p}\right]}^{q}\right\},$$

where $\Delta {H}_{0}$ is a pre-factor related to the kink-pair formation energy, *p* and *q* are fitting parameters, $\tau$ is the applied shear stress and ${\tau }_{0}$ is the Peierls stress. The entropy term $\Delta {S}_{{kp}}$ is simplified and approximated as a constant term by: $\Delta {S}_{{kp}}=\frac{\Delta {H}_{0}}{{T}_{0}}$, where ${T}_{0}$ is a characteristic temperature dependent on the composition of the material. Thus, the free energy of kink-pair formation becomes:

3 $$\Delta {G}_{{kp}}\left(\tau ,T\right)=\Delta {H}_{0}\left\{{\left[1-{\left(\frac{\tau }{{\tau }_{0}}\right)}^{p}\right]}^{q}-\frac{T}{{T}_{0}}\right\}.$$

For the RHEA with local chemical SRO, the nucleation of each kink-pair will break the local order and create a small segment of DAPB, which needs to be included in the Gibbs free-energy term. Here, we add an extra term $\Delta {H}_{{DAPB}}$ to the free energy of kink-pair nucleation, *viz*:

4 $$\Delta {G}_{{kp}}\left(\tau ,T\right)=\Delta {H}_{0}\left\{{\left[1-{\left(\frac{\tau }{{\tau }_{0}}\right)}^{p}\right]}^{q}-\frac{T}{{T}_{0}}\right\}+\Delta {H}_{{DAPB}},$$

where $\Delta {H}_{{DAPB}}=0$ in the RSS sample, and $\Delta {H}_{{DAPB}}$ as a fitting constant for the sample with a fixed amount of SRO. The value of $\Delta {H}_{{DAPB}}$ should scale with ${\gamma }_{{DAPB}}$ and the characteristic length scale of kink-pair nucleus, such as the width and height of the kink-pair nucleus.

In this model, for $\Delta {G}_{{kp}}\left(\tau ,T\right) > 0$, the mobility is dominated by the kink-pair nucleation and migration mechanism. According to Po et al.^52^, the velocity of the screw dislocation can be written as:

5 $$v\left(\tau ,T\right)=\frac{\tau b}{B(\tau ,T)}{\exp }\left(-\frac{\Delta {G}_{{kp}}\left(\tau ,T\right)}{2{k}_{B}T}\right).$$

Here, $B\left(\tau ,T\right)=\frac{a\left[2a\,{\exp }\left(\frac{\Delta {G}_{{kp}}\left(\tau ,T\right)}{2{k}_{B}T}\right)+L\right]}{2{hL}}{B}_{k},$ where $a$ is the lattice parameter, ${k}_{B}$ is the Boltzmann constant, $L$ is the length of the screw dislocation line, $h$ is the height of a kink in the glide direction, and ${B}_{k}$ is a fitted kink drag coefficient. The effect of DAPB during kink-pair migration along the dislocation line is assumed to be small compared to the kink-pair nucleation; it is thus not included in the current model, which leads to a fixed ${B}_{k}$. As the stress and temperature increases, the $\Delta {G}_{{kp}}\left(\tau ,T\right)$ term will decrease and dislocation motion will transfer into a phonon-drag regime, which leads to $v\left(\tau ,T\right)=\tau b/B(\tau ,T)$, with the classic drag coefficients as $B\left(\tau ,T\right)={B}_{0}+{B}_{1}T$, where ${B}_{0}$ and ${B}_{1}$ are fitting parameters. Here, we have neglected the effect of the DAPB strengthening, which will be small relative to the values of *τ* where phonon drag is observed.

In summary, the following phenomenological mobility law for screw dislocation motion is fitted to our simulation results:

6 $$v\left(\tau ,T\right)=\left\{\begin{array}{ll}\frac{\tau b}{B(\tau ,T)}{\exp }\left(-\frac{\Delta {G}_{{kp}}\left(\tau ,T\right)}{2{k}_{B}T}\right)&{if}\Delta {G}_{{kp}}\left(\tau ,T\right) > 0\\ \frac{\tau b}{B(\tau ,T)}&{if}\Delta {G}_{{kp}}\left(\tau ,T\right)\le 0\end{array}\right.$$

7 $$B\left(\tau ,T\right)=\left\{\begin{array}{ll}\frac{a\left[2a\,{\exp }\left(\frac{\Delta {G}_{{kp}}\left(\tau ,T\right)}{2{k}_{B}T}\right)+L\right]}{2{hL}}{B}_{k}\,&{if}\Delta {G}_{{kp}}\left(\tau ,T\right) > 0\\ {B}_{0}+{B}_{1}T\,&{if}\Delta {G}_{{kp}}\left(\tau ,T\right)\le 0\end{array}\right.$$

8 $$\Delta {G}_{{kp}}\left(\tau ,T\right)=\Delta {H}_{0}\left\{{\left[1-{\left(\frac{\tau }{{\tau }_{0}}\right)}^{p}\right]}^{q}-\frac{T}{{T}_{0}}\right\}+\Delta {H}_{{DAPB}},$$

where a sigmoidal function centered around $\Delta {G}_{{kp}}\left(\tau ,T\right)=0$ was used to interpolate the transition smoothly for $v\left(\tau ,T\right)$ and $B\left(\tau ,T\right)$. Details of the fitting procedures are described in Supplementary Note 2 with the values of the fitted parameters listed in Supplementary Table 2.

**Fig. 4:**
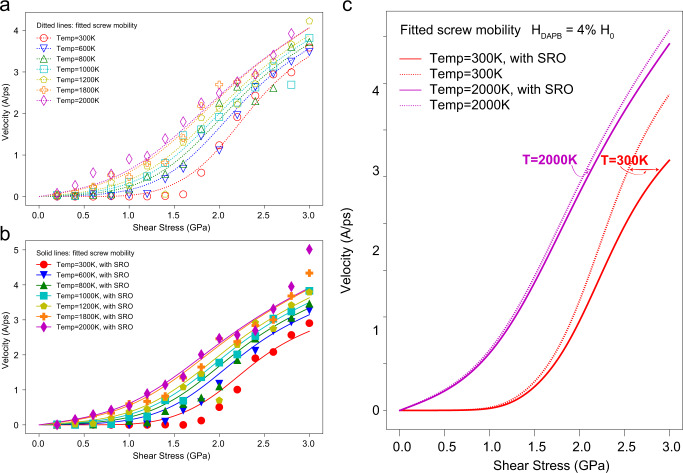
Fitted screw-dislocation mobility model and the influence of thermal entropy on the SRO-strengthening effect. **a** Velocity vs. stress results over a range of temperatures for the screw dislocation in the RSS sample. The open symbols are the simulation data, and the dashed lines are the fits of the phenomenological model described in the text. **b** Velocity vs. stress results over a range of temperatures for the screw dislocation in the systems with SRO. The solid lines are the fitted screw-dislocation mobility based on the phenomenological model with the $\Delta {H}_{{DAPB}}$ term included in the kink-pair formation free energy. **c** The influence of temperature on the SRO strengthening of the screw dislocation. Solid lines represent the fitted model results for the sample with SRO and the dashed lines represent the fitted model for the RSS sample. The strengthening effect due to SRO attenuates when temperature increases from 300 to 2000 K.

The fitted velocity model is plotted with the simulation results in Fig. 4a. We note that the parameters in the phenomenological model used here to fit the MD data, such as kink-pair formation enthalpy, are effective values and do not represent the fact that these quantities will possess a statistical distribution in a RHEA. To investigate the underlying physics of the temperature-dependent SRO effect that we observe from the MD-derived dislocation velocity data, we have included a SRO enthalpic term $\Delta {H}_{{DAPB}}$ in the free energy of kink-pair nucleation, to represent the extra energy required to disrupt the SRO during the kink-pair formation process. In so doing, we assume that the dominant effect of SRO is on the kink-pair formation energy, and neglect its effect on kink motion after formation.

With the fitted parameters from the RSS samples, we keep all other parameters in the model fixed and only fit the $\Delta {H}_{{DAPB}}$ for the SRO samples. Figure 4b shows the fitted model along with the simulation results for the systems with SRO. From the current model, $\Delta {H}_{{DAPB}}$ should be positively correlated with the degree of SRO in the sample; a higher value of $\Delta {H}_{{DAPB}}$ would lead to a higher SRO-strengthening effect. With respect to the temperature-dependence of the SRO effect, the mobility curves based on the model for two temperatures, 300 and 2000 K, are plotted on Fig. 4c, with the dashed lines representing the RSS cases ($\Delta {H}_{{DAPB}}=0$) and the solid lines considering the SRO effect ($\Delta {H}_{{DAPB}}=4 \% \Delta {H}_{0}$). The model also shows that at high temperature (2000 K), the strengthening effect from SRO is significantly diminished compared to behavior at room temperature (300 K), which is consistent with the MD results. Based on the model, we can attribute this attenuation to the thermal entropy term $\Delta {S}_{{kp}}$. With increasing temperature, the contribution from $\Delta {S}_{{kp}}$ to the kink-pair formation free energy (Δ*G*_*kp*_) increases linearly, causing $\Delta {G}_{{kp}}$ to decrease to zero at lower stresses as the temperature is raised. The contribution of $\Delta {H}_{{DAPB}}$ will thus be weakened due to the larger contribution from the entropy term at high temperatures, and this is reflected in the dislocation mobility curves in Fig. 4c.

In summary, in this section, we have fit screw dislocation velocity data, in the absence of the cross-kink pinning, in the MoNbTaW RHEA with a phenomenological model that includes the effect of SRO on the formation energy for a double kink. The strengthening induced by the presence of the local chemical SRO is found to be highly temperature-dependent; specifically, it is found to decrease at high homologous temperatures.

### Edge-dislocation velocities and the influence of SRO

**Fig. 5:**
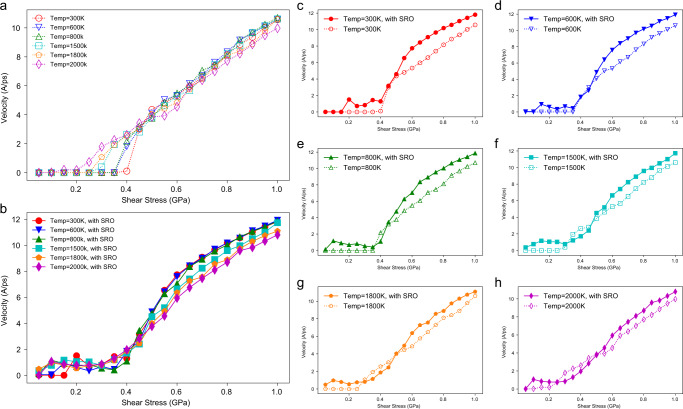
The effect of SRO on the velocities of edge dislocations. **a** Velocity vs. stress results from the simulations of edge dislocations in the RSS sample are shown with open symbols with data for each temperature connected by dashed lines that represent guides to the eye. **b** Similar results as in panel (**a**), but for edge dislocations in the sample with chemical SRO. **c**–**h** Detailed comparisons of the influence of SRO at representative temperatures, from 300 to 2000 K.

We consider the next MD results for edge dislocation motion in both RSS and SRO samples. Figure 5 shows the MD simulation results over a range of temperatures. Figure 5a represents the mobility data in the RSS sample with the stress ranging from 0 to 1.0 GPa, at temperatures from 300 to 2000 K, with Fig. 5b representing results for the sample with SRO. Comparing the behavior of the screw and edge dislocation, it is clear that the velocities of the edge dislocations are much higher than that of the screw dislocations under similar applied stresses. Different from the kink-pair nucleation mechanism in screw dislocations, the glide of edge dislocations in *bcc* metals is generally believed to be governed by solute drag, phonon drag, and wave speed limitation over different velocity regimes^53^.

The influence of SRO on the edge dislocation motion is investigated by comparing the velocity data in Fig. 5a and b. For both low and intermediate stress regimes, the edge dislocation velocities for the system with SRO differ significantly from those in the RSS sample. In the low-stress regime, the glide of an edge dislocation in the RSS sample shows the “friction stress” effect, where the dislocation stays immobile until the applied shear stress exceeds a critical value; this critical stress decreases as the temperature increases. A similar phenomenon has been observed in other alloy systems^47,54,55^, which can be attributed to the pinning of the dislocation due to the ﻿collective interaction of random solute atoms. However, in Fig. 5b, with the presence of SRO, the friction phenomenon is not as effective as in the RSS sample. Instead, the edge dislocation is not immobile but glides at a very low average velocity (<2 $\mathring{\rm A} /{{{{{\rm{ps}}}}}}$) in the low-stress regime (the nonmonotonic behavior displayed by the data in this regime in Fig. 5c–h is likely due to limitations in statistical sampling and is  interpreted as statistical scatter). This is in contradiction with previous understanding^48^ that the onset of dislocation glide necessitates the break-up of local order on the slip plane, which requires extra energy and stress. Consequently, there must be another competing mechanism that enhances the dislocation mobility through the SRO at the same time.

A detailed comparison of edge dislocation velocities at different temperatures in Fig. 5c–h reveals that in the high-stress regime, the influence of SRO on the motion of the edge dislocation is the direct opposite from that for the screw dislocation, in that the velocity of the edge dislocations is enhanced in the SRO sample compared to that in the RSS sample. It is important to note, however, that in the simulations in the high-stress regime, the edge dislocation must glide through the periodic boundary multiple times, such that the SRO on the specific slip plane will have already been destroyed after the edge first crosses through the cell. Nevertheless, although the local slip plane becomes randomized, there is still a significant difference in the velocities of the edge dislocation in the SRO versus the RSS samples, such that we conjecture that the SRO in the neighboring planes could influence the edge dislocation even when the order on the glide plane has been disrupted by dislocation motion. In this case, there is then the question of how the existence of SRO can influence edge dislocation motion and what characteristic length scale is associated with the influence of SRO for an edge dislocation.

One reasonable hypothesis is that the existence of SRO can also influence the solute interactions and, as such, lower the solute drag effect to enhance the mobility of the edge dislocation. From the nature of SRO, the sample with SRO will always have lower configurational energy; furthermore, our previous DFT study showed a flattened energy landscape for the screw dislocation cores in the RHEA due to the presence of SRO^27^, which can be assumed also to be of relevance to edge dislocations. In support of this hypothesis, the initial relaxed edge dislocation line in the SRO sample is observed to be straighter than that in the RSS sample, implying a flatter energy landscape in the former case. Here, the effect of SRO in inducing a considerably flattened energy landscape (lower solute drag effect) could offset the extra energy cost to create a DAPB, such that the dislocation mobility would be enhanced by SRO to give a lower friction effect and higher average velocity.

**Fig. 6:**
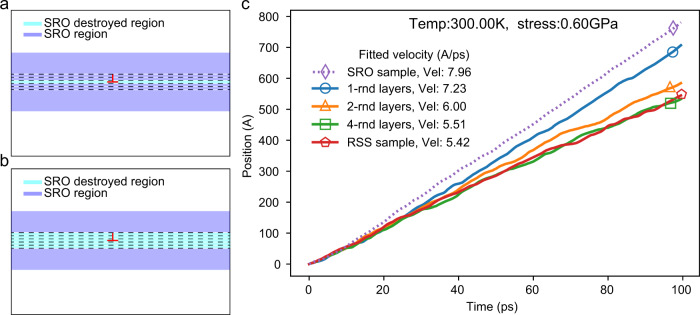
Attenuated drag effect for the edge dislocation induced by SRO in layers neighboring the guide plane. **a** The schematic figure illustrates the state of SRO in the sample. The edge dislocation is represented in red, indicating the position of the glide plane, and the dashed lines are the corresponding atomic planes parallel to the guide plane. The cyan color represents that the SRO on the slip plane has been destroyed after the edge dislocation glides across the whole periodic cell. The blue regions reflect planes where the SRO remains present. **b** This panel represents the situation for simulations in which SRO is destroyed not only along the slip plane but in neighboring parallel planes. **c** The effect of the SRO state around the neighboring slip planes of the edge dislocation motion (distance vs. time). The dashed line represents the average velocity of the edge dislocation in a sample where SRO is destroyed only within the guide plane (corresponding to panel (**a**)). The solid lines represent the edge dislocation motion in the samples with a controlled number of destroyed SRO layers, i.e., for different thicknesses of the cyan region in panel (**b**). Also shown are results obtained for the RSS sample where SRO is not present on any planes in the system.

Accordingly, additional simulations were conducted to further investigate this hypothesis. We show in Fig. 6a, b schematics representing the state of SRO in the sample, where the dashed lines represent the atomic planes, the blue section represents a region where equilibrated SRO is maintained, and the cyan represents regions where the SRO has been destroyed. For the system with SRO, when the edge dislocation crosses through the periodic boundary multiple times, as noted above, the order on its slip plane actually has already been destroyed, but the solute configurations of the atoms in the neighboring regions still display SRO, as shown in Fig. 6a. In addition to comparing with the fully RSS sample, we can manually randomize several layers of atoms around the original slip plane to create an RSS region of limited size. Specifically, if $n$ layers of atoms are randomized, $n+1$ layers of ordering will be destroyed, as illustrated in Fig. 6b. Thus, for the additional simulations, 1, 2, and 4 layers of atoms are manually randomized to create an RSS region of limited size so that the average edge dislocation velocity within these regions can be calculated and compared with the samples with complete SRO and the RSS samples.

Figure 6c shows the position vs. time curves of an edge dislocation in the five different samples described above with an applied 0.6 GPa shear stress at 300 K. The fitted average dislocation velocities are shown in the inset to Fig. 6c. It is observed that the dislocation velocity is highest in the sample with the highest degree of SRO, i.e., only order on the slip plane has been destroyed. With more layers of atoms around the slip plane being randomized, the RSS region becomes thicker and the average dislocation velocity keeps decreasing. When four layers of atoms are randomized in the SRO sample, the thickness of the RSS region is 1.2 nm and the average velocity of the dislocation nearly converges to the velocity in the sample with the complete random disorder. This result supports our hypothesis that SRO would reduce the solute drag interaction for the edge dislocation, and shows that this effect extends to a characteristic length scale of ~1.2 nm. Thus, different from the strengthening effect due to SRO for screw dislocations, the existence of SRO is found to enhance the mobility of edge dislocations in the RHEA.

### Mobility of screw vs. edge dislocations in the MoNbTaW RHEA and pure Mo and Nb metals as a function of temperature

**Fig. 7:**
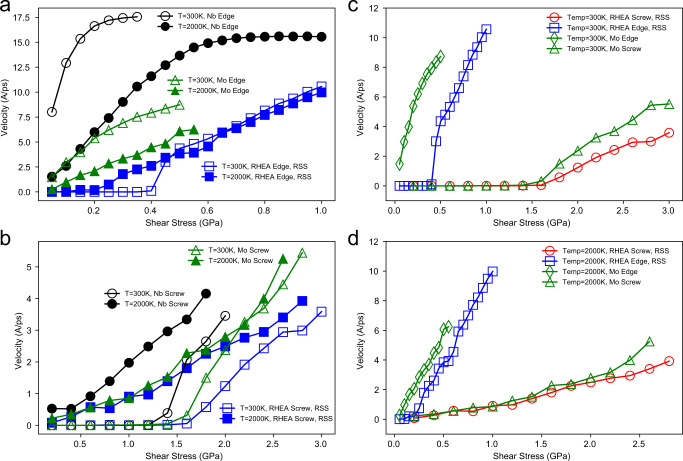
Comparison of dislocation velocities in the MoNbTaW RHEA and pure Mo and Nb. Velocity as a function of stress for **a**, edge dislocations and **b**, screw dislocations in the RHEA and in pure Mo and Nb at temperatures of *T* =  300 and 2000 K. Velocity as a function of stress for edge and screw dislocations in the RHEA and in pure Mo at temperatures of **c**, 300 K and **d**, 2000 K. For all of the results the symbols are simulation data and the solid lines are guides to the eye, connecting results for a given dislocation character (edge or screw) and given temperature.

In addition to the RHEA, the dislocation mobilities in pure Mo and Nb are also calculated for reference, in the same manner, described in “Methods” using the MTP. Figure 7a displays the mobility of the edge dislocation in the RSS RHEA as compared to pure Mo and Nb at temperatures of 300 and 2000 K. The most notable difference in this comparison is that there is no threshold stress effect in the pure metals, associated with solute drag. The mobilities in Mo and Nb show a significant influence of phonon drag, which increases with increasing temperature. The mobility in Mo is much lower than that in Nb but still higher compared to the RSS RHEA. However, this difference in dislocation velocities in the RHEA and Mo diminishes as the temperature is increased; specifically, the velocity difference between the RHEA and pure metal is far smaller at 2000 K than that at 300 K.

Figure 7b shows the velocity results for screw dislocations in the RSS RHEA as compared to those in pure Mo and Nb at temperatures of 300 and 2000 K. As above, we focus on the data for RHEAs where screw dislocation motion is governed by the double-kink nucleation mechanism, excluding the data associated with cross-kink pinning. This motion in both the pure metals and the RHEA displays the friction effect at 300 K, as the glide of the screws in all these materials is dominated by kink-pair nucleation; however, the friction stress disappears at 2000 K in all cases. Similar to the edge dislocation, the velocities of the screw dislocations in the RHEA are lower than those in Mo and Nb at 300 K. However, the difference in screw dislocation velocity in the RHEA and in Mo almost vanishes at this higher temperature. The mobility data of the screw dislocation in tungsten from the previous MD simulation^56^ are also included in Supplementary Fig. 4 for comparison.

Summarizing, at low temperature (300 K) the velocities of both the edge and screw dislocations moving under the double-kink mechanism show significant differences in the RHEA compared to that in the pure *bcc* metals. However, at high temperature (2000 K), the mobility curves of the screw dislocation in the RHEA and pure Mo are almost identical, although the mobility of the edge dislocation in RHEA is still lower than that in the pure metals as it retains some friction stress due to the solute drag effect.

One might ask what the relevance is of such observations. RHEAs have been actively investigated due to their demonstrated high elevated-temperature strength^57^. For pure *bcc* metals, it is well known, e.g., from in situ TEM experiments^49,58^, that edge dislocations play a minor role in strengthening at low and moderate temperatures due to their higher mobility. However, from recent studies for *bcc* RHEAs, the role of the edge dislocations has been suggested to play a more important role in the high-temperature regime^21,26,59^; High energy barriers for edge dislocation motion in RHEA have also been investigated through atomic simulations^60^; additionally, a large fraction of non-screw segments has been observed during the glide of dislocations in such alloys^16^. In this work, we have calculated the velocities of the screw and edge dislocations over a wide range of temperatures in such a RHEA and have shown that the mobility of the edge dislocations does decrease with increasing temperature due to phonon drag; moreover, at the same time, the velocity of the screw dislocations moving by a double-kink mechanism is found to increase with temperature due to a lowering of the free-energy barrier for kink-pair nucleation.

Figure 7c, d shows the representative velocity versus stress curves for edge dislocations and screw dislocations moving by double-kink mechanisms in the MoNbTaW and Mo at 300 and 2000 K. The ratio of the edge-to-screw velocities does indeed decrease as the temperature is raised, as can be seen by the fact that the angle between the velocity versus stress curves for the screw and edge dislocations in MoNbTaW becomes smaller from Fig. 7c, d. The edge-to-screw mobility ratio is around 20 at 300 K, which decreases to 11 when the temperature is raised to 2000 K. However, the mobility of the edge dislocation remains much higher than that of the screw dislocation. Inclusion of the cross-kink mechanism which serves to pin the screw dislocation could further slow the screw dislocations, and further increase this difference. The discrepancy between the current edge-to-screw velocity ratios compared with the CoFeNiTi system studied by Chen et al.^59^ might be due to the different levels of lattice distortion in the two alloy systems. A similar trend in decreasing mobility ratios with increasing temperature is also observed in pure Mo, as shown in Fig. 7c, d, which indicates that this is not a unique feature of RHEAs.

One significant temperature-dependent property that differentiates the mobility of screw and edge dislocations, which is also a unique property in the RHEAs, is the enhanced friction stress. For edge dislocations, the friction is zero in pure metals for edge dislocations, whereas due to solute drag interactions we can clearly observe temperature-dependent friction stress for the edge in the RHEA, as shown in Figs. 5a and 7. For screw dislocations, the friction phenomenon is due to the energy barrier associated with kink-pair nucleation, which holds for both pure metals and RHEAs. Accordingly, the different mechanisms of the friction effect for edge and screw dislocations naturally lead to different dependences of the critical friction stress on temperature. Here, the critical friction stress is determined as the stress for which the velocity reaches 0.2 $\mathring{\rm A} /{{{{{\rm{ps}}}}}}$ for screw dislocations and 1.0 $\mathring{\rm A} /{{{{{\rm{ps}}}}}}$ for edge dislocations.

**Fig. 8:**
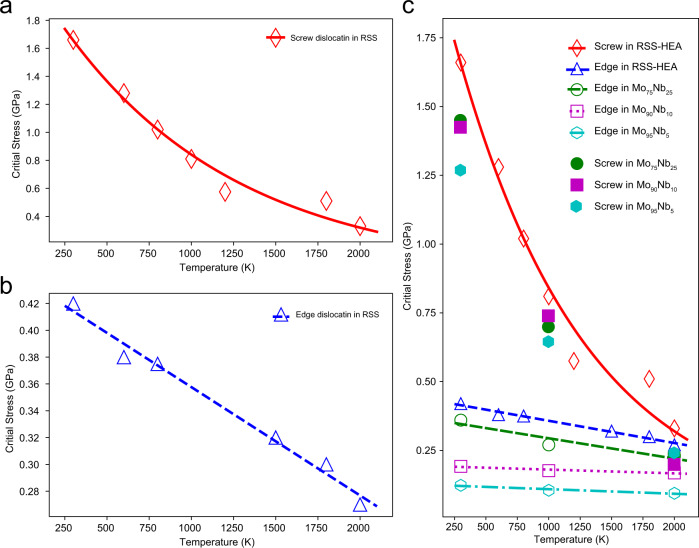
Critical friction stress as a function of temperature for screw and edge dislocations. **a** Critical stress as a function of temperature for the screw dislocation in RSS samples obtained from MD simulations (open symbols), fitted with an exponential function (solid line). **b** Critical stress as a function of temperature for the edge dislocation in RSS samples obtained from MD simulations (open symbols), fitted with a linear function (solid line). **c** Comparison of critical stresses for screw and edge dislocations as a function of temperature. The critical stresses for edge and screw dislocations in Mo–Nb binary alloys are included for comparison.

Figure 8a shows the critical stress as a function of temperature for the screw dislocation in the RSS RHEA, which are fit well by an exponential decay curve, whereas in Fig. 8b, the critical stress for the edge dislocation is well fit by a function that decreases linearly with temperature. Moreover, the critical stress for the screw dislocation is more sensitive to the increment of temperature and, as such, decays at a much higher rate compared with the edge dislocation. According to our simulation data, from 300 to 2000 K the critical friction stress decreases by 140 MPa for the edge dislocation, whereas the decrease is almost an order of magnitude larger at 1.3 GPa for the screw dislocation. Although the initial friction is much higher for the screw dislocation at room temperature, the critical stresses for both the screw and edge decay to approximately the same level at ~2000 K (Fig. 8c).

For comparison and to further show the unique features of the RHEA, we also calculated friction stresses of some binary alloys through the same procedures, from Mo_95_Nb_5_ to Mo_75_Nb_25_. Figure 8c shows that the overall values of the critical friction stresses in the binary alloys are lower compared with the RHEA. Moreover, the slope of the critical stress for the edge dislocation is also decreasing at lower solute concentrations, where the friction stress is expected to converge to a horizontal line with zero friction stress in the pure *bcc* metal for edge dislocation motion. However, due to the kink nucleation barrier, the screw dislocation will still maintain an observable level of critical friction stress in the pure *bcc* metal.

In summary, we have developed a new machine-learning interatomic potential and used it as the basis for systematic MD studies of the mobility of edge and screw dislocations over a wide range of temperatures in a *bcc* MoNbTaW refractory high-entropy alloy (RHEA).

- Short-range order (SRO) is found to play opposite roles in the motion of screw and edge dislocation in RHEAs: it will hinder screw dislocation motion yet accelerate the motion of edges.
- A novel cross-slip locking mechanism is observed in the RHEA even with limited cell size, which can provide extra strengthening to the RHEA.
- A different temperature sensitivity for the critical stress to initialize dislocation motion is found only in the RHEA, but not in elemental *bcc* metals. The critical friction stress for the edge dislocation decays much slower than it does for the screw dislocation with increasing temperature, and it becomes comparable in magnitude for both edge and screws at 2000 K.

In detail, screw dislocations in the MoNbTaW RHEA move via kink-pair nucleation and migration. The cross-slip locking mechanism observed in the simulations can be attributed to the intrinsic fluctuation of energy along the dislocation line and the rugged energy landscape in the system; short-range order (SRO) is found to induce a temperature-dependent strengthening effect on the screw dislocations moving through the kink-pair nucleation mechanism while accelerating the edge dislocation movement through attenuation of the magnitude of the solute interactions. A phenomenological model for the mobility of screw dislocations moving via a double-kink mechanism has been constructed to reveal the origin of the temperature-dependent SRO effect for screw dislocation motion; this mechanism arises from the contribution of SRO to the free-energy barrier for kink-pair nucleation. With increasing temperature, a reduced edge-to-screw velocity ratio was discovered in both pure *bcc* metals and in the MoNbTaW RHEA.

These findings demonstrate salient mechanisms and specific influences of temperature and local chemical order on these mechanisms of edge and screw-dislocation motion in RHEAs, all of which can have a significant impact on the mechanical properties of RHEAs.

## Methods

### Machine-learning interatomic potential

The MTP model and its formalism have been extensively studied and applied in the previous work^44,61,62^. The MTP essentially constructs contracted rotationally invariant local environment descriptors for each atom in the system and builds a polynomial regressed correlation between the potential energy surface (PES) and these descriptors. The descriptors, named moment tensors, are devised as follows:

9 $${M}_{\mu ,\nu }({R})={\sum }_{j}{f}_{\mu }(|{{{R}}}_{ij}|,{z}_{i},{z}_{j})\underbrace{{R}_{ij}\otimes \cdots \otimes {R}_{ij}}_{{\nu} \,{{{{{\rm{times}}}}}}},$$

where the functions ${f}_{\mu }$ are the radial distribution of local environment around atom $i$ and these functions are specified according to the atomic type of the neighbor atom $j$. The terms ${{{{{{\boldsymbol{R}}}}}}}_{{ij}}\bigotimes \cdots \bigotimes {{{{{{\boldsymbol{R}}}}}}}_{{ij}}$ are tensors of rank $\nu$ encoding the angular information about the local environment. The basis functions $B({{{{{\boldsymbol{R}}}}}})$ are formulated by contracting the moment tensors ${M}_{\mu ,\nu }$ to a scalar, and the potential energy of atom $i$ are linearly expanded on a set of the basis functions:

10 $${E}_{i}\left({{{{{\boldsymbol{R}}}}}}\right)=\mathop{\sum}\limits_{l}{\beta }_{l}B\left({{{{{\boldsymbol{R}}}}}}\right).$$

There are two key hyperparameters that determine the performance of the accuracy and computational cost of the MTP: the radius cutoff (${R}_{{{{{{\rm{cut}}}}}}}$) and maximum level (${{lev}}_{\max }$). The ${R}_{{{{{\rm{cut}}}}}}$ encodes the extent of atomic interactions in the local environment, and the ${{lev}}_{\max }$ controls the completeness of the basis functions $B({{{{{\boldsymbol{R}}}}}})$, which in turn influences the computational cost and likelihood of over-fitting. In this work, we adopted the grid search method to examine the choice of hyperparameters by evaluating the performance on reproducing the basic materials properties, e.g., elastic constants and stacking fault energies. The ${R}_{{{{{{\rm{cut}}}}}}}$ and ${{lev}}_{\max }$ were set to 4.7 Å and 14, respectively. The energy and force data were assigned weights of 1 and 0.01, respectively, in line with previous studies^46,63^.

### Local chemical short-range order parameter

Instead of the more generalized ordering parameter developed by Ceguerra^64^, we adopted a simpler pairwise multicomponent SRO parameter, specifically the Warren Cowley order parameter^65^, ${\alpha }_{{ij}}=1-\frac{{P}_{j,i}}{{c}_{j}}$, to quantify the SRO in each specific nearest-neighboring shell. ${P}_{j,i}$ is the fraction of species *j* in the nearest-neighboring shell around *i*, and ${c}_{j}$ is the concentration of *j*. To indicate the overall degree of SRO, we make use of a quantity given as the sum of all the $\left|{\alpha }_{{ij}}\right|$ for all species at the nearest-neighbor shell (${SRO}={\sum }_{i,j}\left|{\alpha }_{{ij}}\right|$).

### Molecular dynamic simulation

MD simulations were performed using the software package LAMMPS^66^ and the atomic configurations were displayed by OVITO^67^. The simulation cells are illustrated in Supplementary Fig. 5, with dimensions of ~150 Å in *x*, 320 Å in *y*, 200 Å in *z,* and contains 573,672 atoms.

#### MC/MD simulation

The MC/MD simulations were first conducted to equilibrate the SRO before the dislocation line was inserted in the cell. The periodic boundary conditions were set for all three directions. The samples were initially relaxed and equilibrated at 800 K and zero pressure under the NPT ensemble through MD. After that, MC steps consisting of attempted atom swaps were conducted, hybrid with the MD. In each MC step, a swap of one random atom with another random atom of a different type was conducted based on the Metropolis algorithm in the canonical ensemble. 10^2^ MC swaps were conducted at every 10^3^ MD steps with a time step of 0.001 ps during the simulation.

#### Dislocation mobility simulation

Similar to previous MD studies of dislocation mobility in pure *bcc* metals^68–70^, the dislocation line was inserted in the simulation cell based on the Burgers vector^47^ for both RSS and the SRO samples after MD/MC relaxation. Periodic boundary conditions were imposed along *x*- and *y* directions with a shrink-wrapped nonperiodic boundary condition in *z* direction. The samples were initially relaxed and equilibrated at the targeted temperature with pressure in the *x*- and *y* directions equilibrated to zero through the NPT ensemble for 50 ps. During the loading step, the bottom two layers of atoms in the *z* direction were fixed and the top two layers of atoms were treated as a rigid body with different levels of shear stresses applied, while the NVT ensemble was applied to other atoms. To minimize the effect of stress waves and oscillation of the shear stress, the applied shear stress was gradually ramped^71^ from zero at a rate of 20 MPa per 1.5 ps. When the shear stress reached the target value, it was held constant for 150 ps while the dislocation positions were recorded.

## Supplementary information

Supplementary Information

Description of Additional Supplementary Files

Supplementary Movie 1

## Data Availability

The data that support the findings of this study are available from Dr. Sheng Yin (email: shengyin@berkeley.edu) upon reasonable request. The parameter files of the potential will be published upon the publication of this manuscript.

## Code availability

All simulations were executed using open-source software LAMMPS. All the other codes that support the findings of this study are available from Sheng Yin (email: shengyin@berkeley.edu) upon reasonable request.
