# Supplementary material for: Atomistic simulations of dislocation mobility in refractory high-entropy alloys and the effect of chemical short-range order
Source: Nat Commun. 2021 Aug 11;12:4873. doi: 10.1038/s41467-021-25134-0 (PMC8357793; doi:10.1038/s41467-021-25134-0)
Supplement: Supplementary file 1 — Supplementary Information [file 41467_2021_25134_MOESM1_ESM.pdf]

Supplementary Information

Atomistic simulations of dislocation mobility in refractory high-entropy alloys and the effect of chemical short-range order

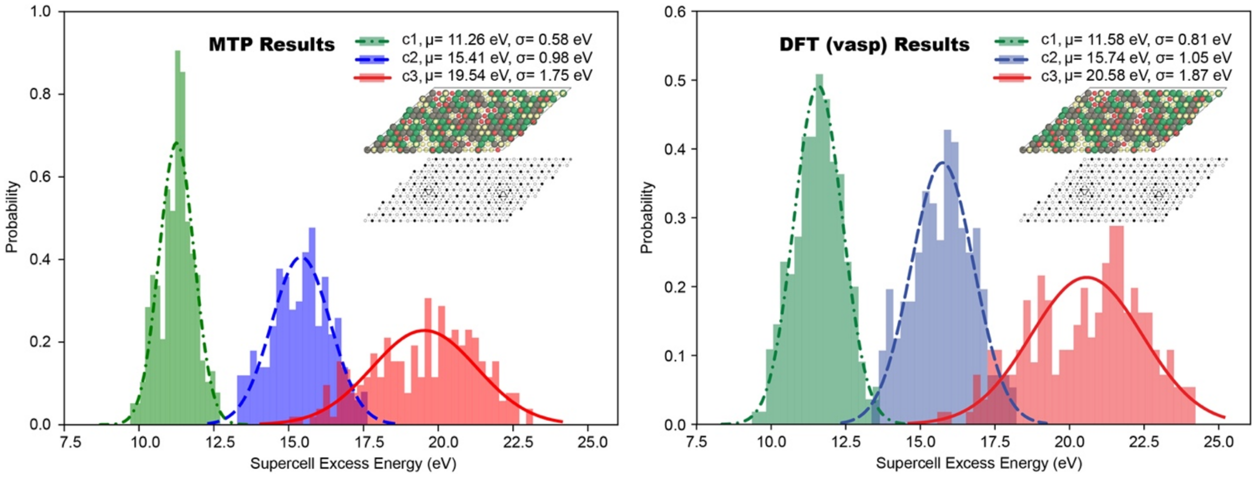

Supplementary Figure 1 | Comparison of dislocation dipole energies calculated from MTP and DFT. Distributions of dislocation dipole energies for varying local environments, calculated through the MTP potential and compared with DFT data. The results were obtained by inserting a dislocation dipole into supercells with three different levels of randomness: c1, c2 and c3. Each configuration contains 231 different individual samples. For the DFT results, the positions of the atoms are relaxed through DFT. For the MTP results, these positions are relaxed in MD based on the MTP forces using the DFT relaxed cells. The histogram of supercell excess energies of different configuration calculated by MD and DFT are plotted in green, blue and red, and fitted with a Gaussian distribution, where μ is the average value and σ is the standard deviation. The results calculated by MTP agree well with the DFT results. (The supercell excess energy is defined as the energy difference between the supercell with and without the dislocation dipole. The detailed dipole cell construction and VASP parameters for the DFT calculations are described in previous work by Yin *et al.*[1])

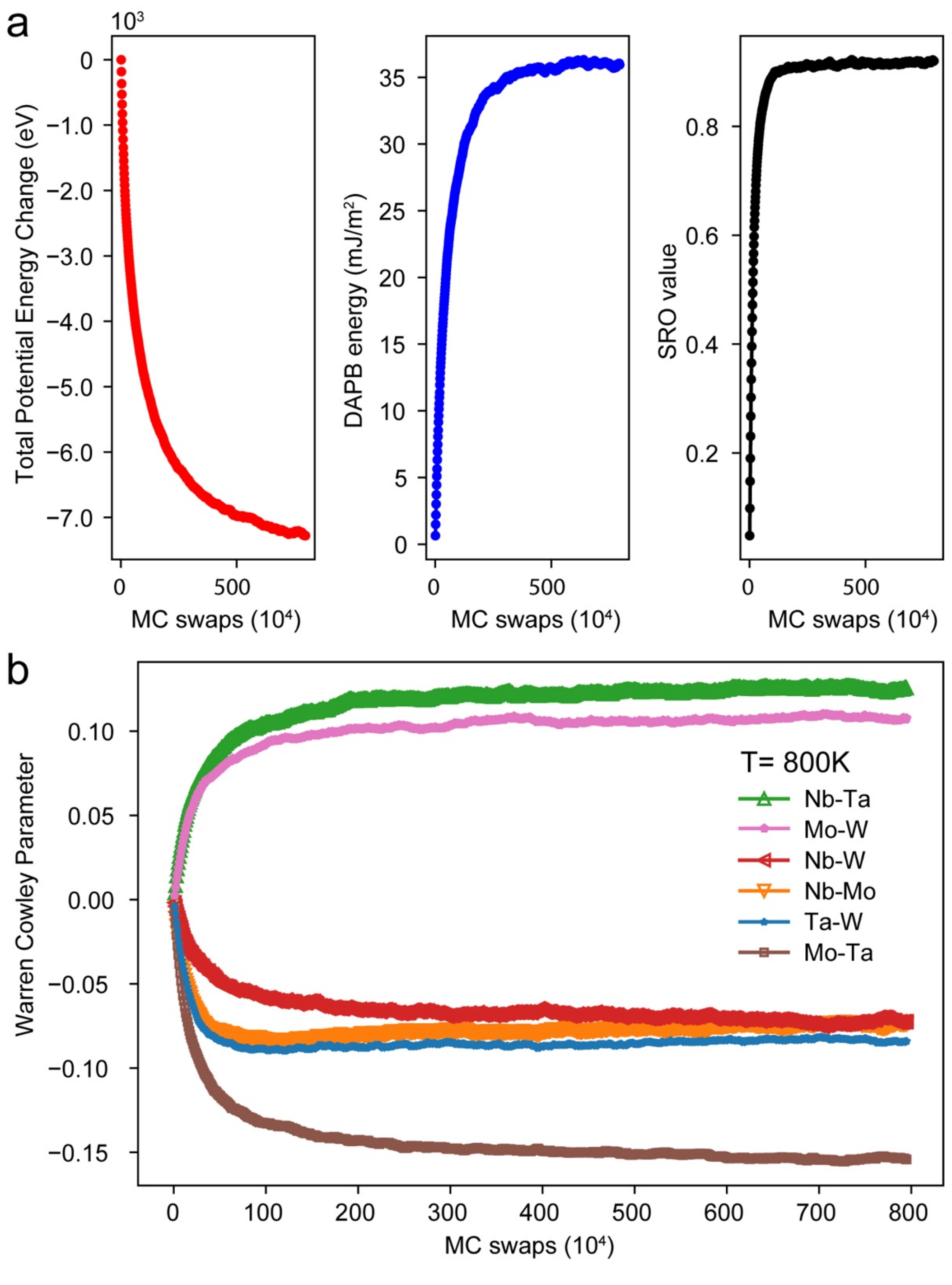

Supplementary Figure 2 | Evolution of potential energy, diffuse antiphase boundary (DAPB) energy and local chemical SRO in the MoNbTaW RHEA from hybrid MC/MD simulations. a, Change in the total potential energy of the simulation cell containing 0.5 million atoms, DAPB energy and cumulative SRO parameter (see Method section) as a function of the number of MC swaps during equilibration at 800K. **b,** The detailed values of the Warren Cowley parameters α_{ij} for different pair types as a function of the MC swaps at the same temperature.

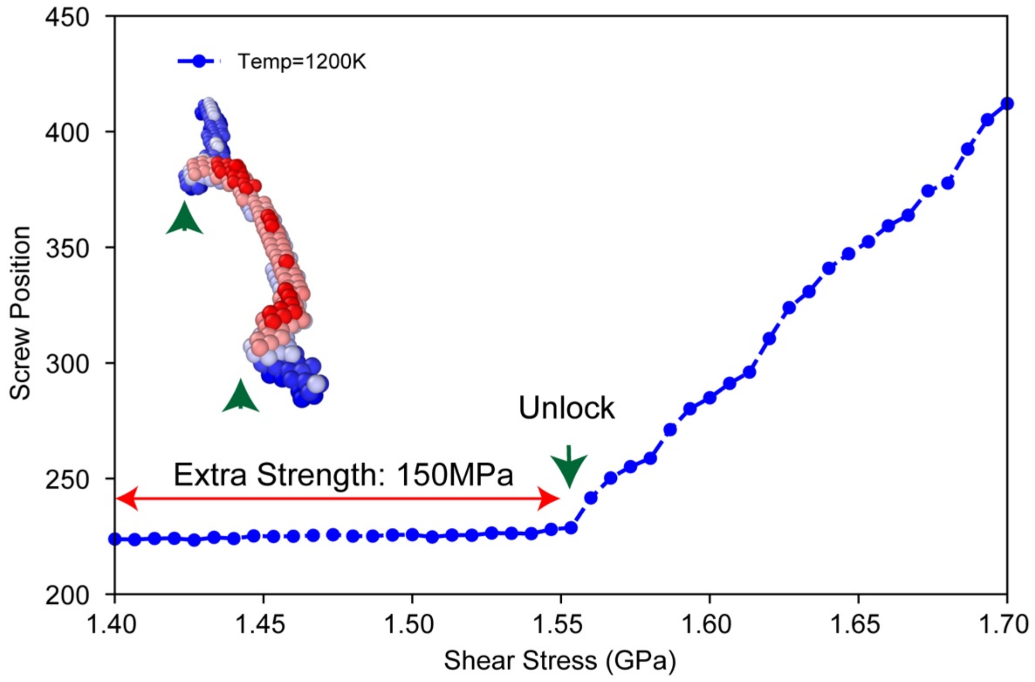

Supplementary Figure 3 | Extra stress required to break the interlocking kinks. Restart from the final configuration shown in Figure 3c, the applied shear stress is increasing at the rate of 1.2 MPa/ps. The screw dislocation stays immobile until the shear stress reaches approximately 1.55 GPa, and the interlocking kinks are unlocked, as shown by the green arrow.

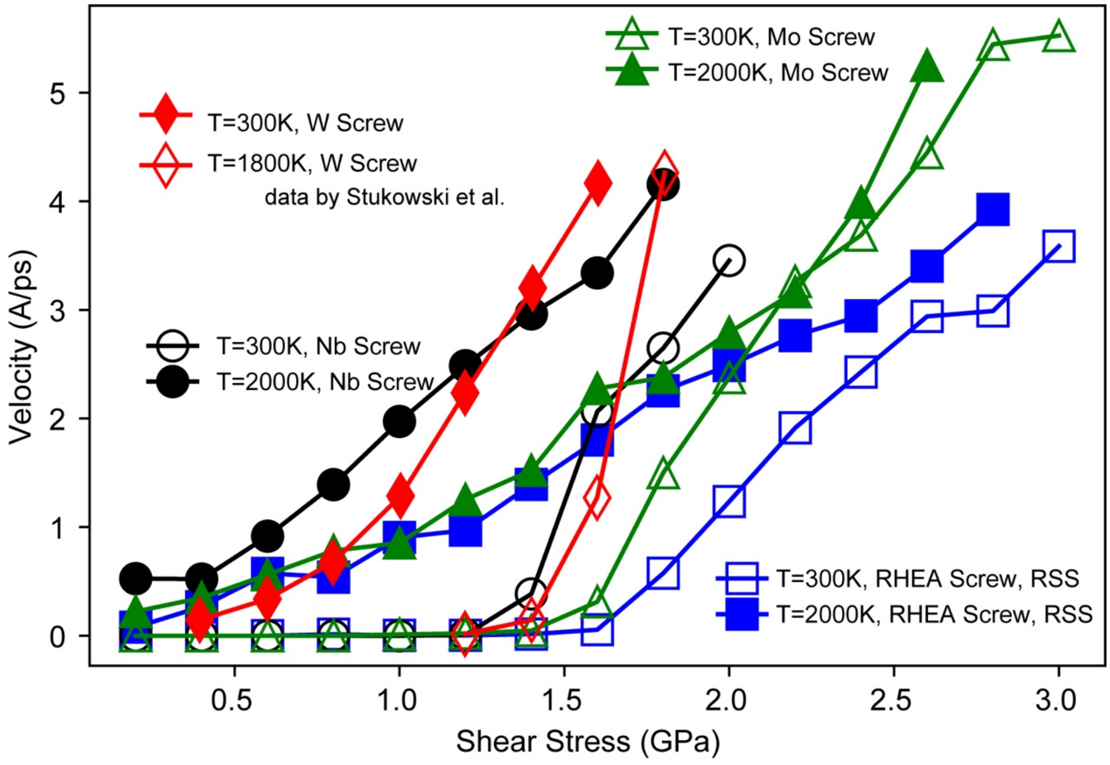

Supplementary Figure 4 | Velocity versus shear stress of screw dislocations in the RHEA and in pure Mo and Nb at temperatures of *T* = 300K and 2000K. Data are compared with corresponding results for screw dislocations in W taken from ref. 2.

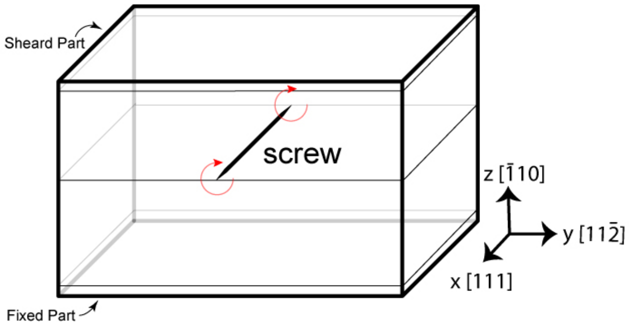

Supplementary Figure 5 | MD Simulation cell for dislocation mobility simulations. Crystalline orientations of the cells are shown, with the screw and edge dislocations gliding on the $(\bar{1}10)$ plane.

Supplementary Table 1 | Basic materials property predictions of the MTP model. The predicted melting points (T_m), unstable stacking fault energy γ_{us} of the (011) γ surface along the $[\bar{1}\bar{1}1]$ direction, elastic constants (c_{ij}), and Voigt-Reuss-Hill[3] bulk modulus (B_{VRH}) for *bcc* Nb, Mo, Ta, and W are compared with DFT and experimental values.

	T_m (K)	γ_{us} (mJ/m^2)	c_{11} (GPa)	c_{12} (GPa)	c_{44}(GPa)	B_{VRH} (GPa)
**Nb**						
Expt.[4]	2750	—	247	135	29	172
DFT	—	827	249	135	19	173
MTP	2700	789 (-4.6%)	238 (-4.4%)	159 (17.8%)	24 (26.3%)	185 (6.9%)
**Mo**						
Expt.[5]	2896	—	479	165	108	270
DFT	—	1468	472	158	106	263
MTP	2860	1256 (-14.4%)	390 (-17.4%)	174 (10.1%)	85 (-19.8%)	246 (-6.5%)
**Ta**						
Expt.[6]	3290	—	266	158	87	194
DFT	—	835	264	161	74	195
MTP	3040	809 (-3.1%)	257 (-2.7%)	161 (0.0%)	67 (-9.5%)	193 (-1.0%)
**W**						
Expt.[7]	3695	—	533	205	163	314
DFT	—	1661	511	200	142	304
MTP	3680	1557 (-6.3%)	480 (-6.1%)	187 (-6.5%)	122 (-14.1%)	285 (-6.3%)

Supplementary Table 2 | Fitted parameters in the phenomenological dislocation mobility model.

	ΔH_0 (eV)	B_k (Pa · s)	B_0 (Pa · s)	B_1 (Pa · s/K)	p	q	ΔH_{DAPB} (eV)
**RSS**	0.346	1.40×10^{-2}	6.17×10^{-12}	3.13×10^{-7}	2.48	6.65	0.0
**SRO**	0.346	1.40×10^{-2}	6.17×10^{-12}	3.13×10^{-7}	2.48	6.65	0.0139

Supplementary Note 1 | Local chemical short-range order in MoNbTaW RHEA.

To attain a significant degree of SRO in the simulation cell, a temperature (T) of 800K was set for the MC simulations, which is close to the order-disorder transition temperature found in previous studies on the MoNbTaW alloys[7,8]. The MC/MD simulation cell was initiated with an RSS configuration and the entire simulation considers approximately 8 million swaps for the cell with a half million atoms, leading to the evolution of the potential energy change shown in Supplementary Fig. 2a. With the development of SRO in the alloys, the presence of dislocation slip can break favorable bonds on the slip plane such that an increase in energy will result through the formation of a diffuse antiphase boundary (DAPB). The magnitude of the DAPB is dependent on the level of SRO and can be quantified through the so-called DAPB energy per unit area (γ_{DAPB}). The γ_{DAPB} as well as the sum of the SRO parameters over distinct pairs are plotted in Supplementary Fig. 2a as a function of the number of MC swaps. The system clearly develops a significant degree of SRO, and the value of γ_{DAPB} equilibrates to ~36 $\mathrm{mJ/m^2}$ on the $[\bar{1}10]$ plane. Supplementary Fig. 2b shows the Warren Cowley parameters[9] for the different pair types, which indicate the preferred type of atomic pairings in the SRO states after the MC/MD relaxation. The chemical preferences for forming like versus unlike pairs appear to be consistent with the state of SRO calculated in previous studies[1,7,8,10]. Specifically, in the equilibrium state of SRO, the dominant effects are an enhancement in the number of Mo-Ta pairs and a reduction in the number of Nb-Ta pairs, compared to the RSS.

Supplementary Note 2 | Phenomenological dislocation mobility model

The phenomenological model for the velocity of screw dislocations moving by the double-kink mechanism is first fit with to the simulated dislocation velocity data from the RSS samples for the unknown parameters ΔH_0, B_k, B_0, B_1, τ_0, p and q, with the fixed values for the following parameters: $T_0 = 0.8T_{melting} = 2541\text{K}$, $\tau_0 = 4$ GPa, $a = 3.22$ Å, $L = 150$ Å and $\Delta H_{DAPB} = 0$. In the fitting process, the sum of the squares of the residuals is minimized through the bounded *fminsearch* function in MATLAB, with the data points of cross-slip locking shown in Fig. 3 excluded. The resulting fitted parameters for the random solid-solution (RSS) RHEA are shown in Supplementary Table 2. With the fitted parameters from the RSS samples, we keep all other parameters in the model fixed and only fit the ΔH_{DAPB} for the SRO samples. The fitting yields $\Delta H_{DAPB} = 0.0139$ eV, which is around 4% of the magnitude of ΔH_0. We can also estimate a value for ΔH_{DAPB}, based on the calculated value of γ_{DAPB} and the geometry of nucleated kink-pair nucleus, in order to compare with the fitted result. The smallest kink-pair width $l^* = \frac{a}{2/\sqrt{3}}$ and the height of a kink $h = \frac{\sqrt{6}}{3}a$ give $\Delta H^*_{DAPB} = \gamma_{DAPB} l^* h = 0.0169$ eV, which is very close to the fitted value.

Supplementary References

1 Yin, S., Ding, J., Asta, M. & Ritchie, R. O. Ab initio modeling of the energy landscape for screw dislocations in body-centered cubic high-entropy alloys. *npj Computational Materials* **6**, 1-11 (2020).

2 Stukowski, A., Cereceda, D., Swinburne, T. D. & Marian, J. Thermally-activated non-Schmid glide of screw dislocations in W using atomistically-informed kinetic Monte Carlo simulations. *International Journal of Plasticity* **65**, 108-130 (2015).

3 Hill, R. The elastic behaviour of a crystalline aggregate. *Proceedings of the Physical Society. Section A* **65**, 349 (1952).

4 Trivisonno, J., Vatanayon, S., Wilt, M., Washick, J. & Reifenberger, R. Temperature dependence of the elastic constants of niobium and lead in the normal and superconducting states. *Journal of Low Temperature Physics* **12**, 153-169 (1973).

5 Simmons, G. Single crystal elastic constants and calculated aggregate properties. (Southern Methodist Univ Dallas Tex, 1965).

6 Featherston, F. H. & Neighbours, J. Elastic constants of tantalum, tungsten, and molybdenum. *Physical Review* **130**, 1324 (1963).

7 Fernández-Caballero, A., Wróbel, J., Mummery, P. & Nguyen-Manh, D. Short-range order in high entropy alloys: Theoretical formulation and application to Mo-Nb-Ta-VW system. *Journal of Phase Equilibria and Diffusion* **38**, 391-403 (2017).

8 Kostiuchenko, T., Körmann, F., Neugebauer, J. & Shapeev, A. Impact of lattice relaxations on phase transitions in a high-entropy alloy studied by machine-learning potentials. *npj Computational Materials* **5**, 1-7 (2019).

9 Cowley, J. An approximate theory of order in alloys. *Physical Review* **77**, 669 (1950).

10 Huhn, W. P. & Widom, M. Prediction of A2 to B2 phase transition in the high-entropy alloy Mo-Nb-Ta-W. *JOM* **65**, 1772-1779 (2013).
