# Supplementary material for: Atomistic simulations of dislocation mobility in refractory high-entropy alloys and the effect of chemical short-range order
Source: Nat Commun. 2021 Aug 11;12:4873. doi: 10.1038/s41467-021-25134-0 (PMC8357793; doi:10.1038/s41467-021-25134-0)
Supplement: Supplementary file 2 — Description of Additional Supplementary Files [file 41467_2021_25134_MOESM2_ESM.pdf]

Description of Additional Supplementary Files

Supplementary Movie 1. Detailed process of the cross-slip locking formation and self-unlocking during the simulation of screw dislocation motion. Only the non-*bcc* atoms are shown and are colored by the coordinates in the $[\bar{1}10]$ direction. The green line represents the screw dislocation line, which is extracted through the Dislocation Extraction Algorithm (DXA) in OVITO.
